# Geometric accuracy of an acrylonitrile butadiene styrene canine tibia model fabricated using fused deposition modelling and the effects of hydrogen peroxide gas plasma sterilisation

**DOI:** 10.1186/s12917-020-02691-y

**Published:** 2020-12-09

**Authors:** Chi-Pin Hsu, Chen-Si Lin, Chun-Hao Fan, Nai-Yuan Chiang, Ching-Wen Tsai, Chun-Ming Chang, I-Li Liu

**Affiliations:** 1 High Speed 3D Printing Research Center, National Taiwan University of Science and Technology, Taipei, Taiwan; 2 Department and Graduate Institute of Veterinary Medicine, School of Veterinary Medicine, National Taiwan University, Taipei, Taiwan; 3 Institute of Veterinary Clinical Science, School of Veterinary Medicine, National Taiwan University, Taipei, Taiwan; 4 National Applied Research Laboratories, Taiwan Instrument Research Institute, Hsinchu, Taiwan

**Keywords:** Fused deposition modelling, Long bone model, Sterilisation, Surgical guide, Three-dimensional printing

## Abstract

**Background:**

Three-dimensional (3D) printing techniques have been used to produce anatomical models and surgical guiding instruments in orthopaedic surgery. The geometric accuracy of the 3D printed replica may affect surgical planning. This study assessed the geometric accuracy of an acrylonitrile butadiene styrene (ABS) canine tibia model printed using fused deposition modelling (FDM) and evaluated its morphological change after hydrogen peroxide (H_2_O_2_) gas plasma sterilisation. The tibias of six canine cadavers underwent computed tomography for 3D reconstruction. Tibia models were fabricated from ABS on a 3D printer through FDM. Reverse-engineering technology was used to compare morphological errors (root mean square; RMS) between the 3D-FDM models and virtual models segmented from original tibia images (3D-CT) and between the models sterilised with H_2_O_2_ gas plasma (3D-GAS) and 3D-FDM models on tibia surface and in cross-sections at: 5, 15, 25, 50, 75, 85, and 95% of the tibia length.

**Results:**

The RMS mean ± standard deviation and average positive and negative deviation values for all specimens in E_FDM-CT_ (3D-FDM vs. 3D-CT) were significantly higher than those in E_GAS-FDM_ (3D-GAS vs. 3D-FDM; *P* < 0.0001). Mean RMS values for E_FDM-CT_ at 5% bone length (proximal tibia) were significantly higher than those at the other six cross-sections (*P* < 0.0001). Mean RMS differences for E_GAS-FDM_ at all seven cross-sections were nonsignificant.

**Conclusions:**

The tibia models fabricated on an FDM printer had high geometric accuracy with a low RMS value. The surface deviation in E_FDM-CT_ indicated that larger errors occurred during manufacturing than during sterilisation. Therefore, the model may be used for surgical rehearsal and further clinically relevant applications in bone surgery.

**Graphical abstract:**

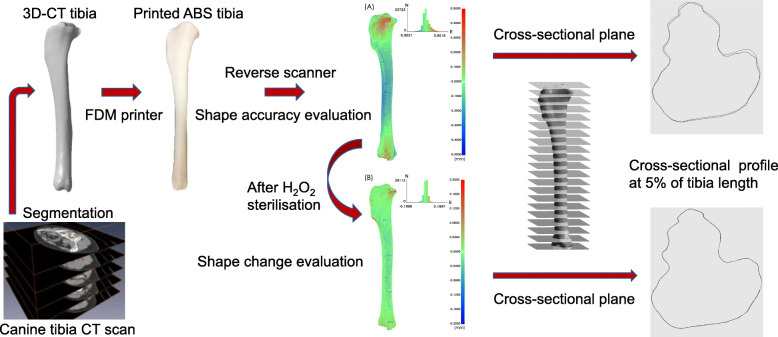

## Background

Three-dimensional (3D) printing techniques have been used extensively for anatomical models, presurgical planning, surgical guiding instruments, custom-made implants, and client communication in human medicine, particularly in orthopaedic, facial reconstructive, spinal, and dental surgery [1–3]. Presurgical planning using 3D-printing anatomical models has been adopted in veterinary medicine [4–6] and related research [7, 8], particularly in orthopaedic surgery, thus improving surgeon confidence and reducing surgical time, the occurrence of surgical complications, and surgeons’ exposure to radiation [4, 6, 8]. These studies have indicated that 3D-printed models are beneficial when used for managing complex cases and when used by inexperienced surgeons [4, 6, 9]. Additionally, 3D-printed bone models are suitable for presurgical rehearsals of plate contouring, osteotomy, and the design of surgical guides [4, 6, 7, 9]. However, in veterinary orthopaedics, only three studies have evaluated the linear deviation of 3D-printed long-bone models [10–12]. Moreover, the geometric accuracy of 3D-printed long bones, which is crucial for bone-plate contouring and the design of patient-specific surgical guides, has not been evaluated.

The majority of commercially available desktop printers for rapid prototyping are based on fused deposition modelling (FDM). These types of desktop printers comprise 46% of all 3D printing devices in use [13]. They are easy to use and suitable for offices, providing prints of similar accuracy to industrial printers [14, 15]. Consumer-grade FDM printers have been used in some studies to produce inexpensive maxillofacial and long-bone models in an office setting [11, 12]. Other studies have demonstrated that 3D-printing of patient-specific guides is a safe, promising, and affordable method that achieves satisfactory clinical outcomes [16, 17]. Even though the plastic filament used in 3D printing has a low melting point, given the printed models intraoperative usage, sterilisation of plastic models using an autoclave has been suggested [3, 12]. Studies, however, have demonstrated deformation of plastic models after steaming [18–20] or alteration to their mechanical strength after gas plasma sterilisation [21]. This evidence suggests such plastic filaments are problematic since they are sensitive to conventional thermal steam sterilisation. However, little is known regarding the accuracy and morphological changes of these plastic bone models after sterilisation. Because this is a new field, further research is warranted [1].

Designing patient-specific surgical tools for canine tibial plateau levelling osteotomy (TPLO), including 3D-printed tibias and bone-cutting instruments, is our field of interest in veterinary medicine. Tibial bone–surface curvature is critical for plate implantation and design of bone-cutting instruments. To the best of our knowledge, no data are currently available regarding the effects of low-temperature sterilisation on the surface accuracy of 3D-printed ABS canine tibias manufactured using FDM. Therefore, an accurate 3D plastic model is crucial for preoperative planning and intraoperative applications, and the extent of plastic model deformation after sterilisation needs evaluation. We focused on hydrogen peroxide (H_2_O_2_) low-temperature sterilisation, which is suitable for surgical instruments that are sensitive to heat and moisture [22].

The objectives of this study were (1) to compare the geometric accuracy of an ABS tibia model 3D printed using FDM with that of a 3D tibia model derived using computed tomography (CT) and (2) to evaluate possible geometric change of the 3D ABS tibia model after sterilisation with H_2_O_2_ gas plasma.

## Results

### Geometric deviation of the whole-tibia model

**Fig. 1:**
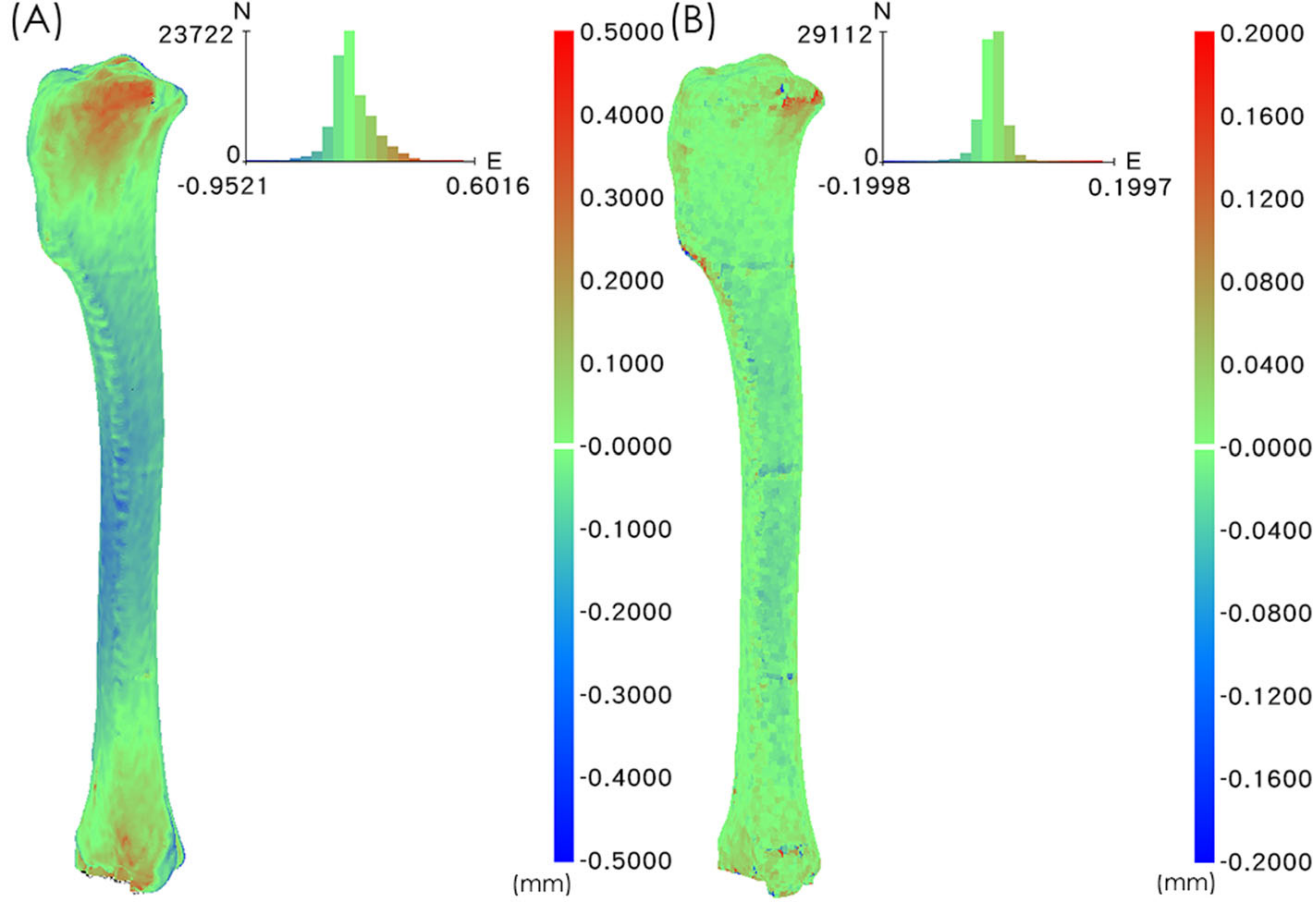
The colour difference map represents the difference between the tibial bone models. **a** Superposition of 3D**-**FDM and 3D-CT tibia models in case No.3. The maximum and minimum values of the colour difference map were + 0.5000 and − 0.5000 mm. **b** Superposition of 3D-GAS and 3D-FDM tibia models in case No.3. The maximum and minimum values of the colour difference map were + 0.2000 and − 0.2000 mm

Table 1 and Fig. 1 provide superimposition results of the RMS values for 12 tibias in the two comparison groups: E_FDM-CT_ (3D-FDM vs. 3D-CT) and E_GAS-FDM_ (3D-GAS vs. 3D-FDM). The RMS mean ± standard deviation and average positive and negative deviation values for all specimens in E_FDM-CT_ (0.1214 ± 0.0185 mm, 0.0973 ± 0.0182 mm, and − 0.0737 ± 0.0170 mm, respectively) were significantly higher than those in E_GAS-FDM_ (0.0431 ± 0.0248 mm, 0.0234 ± 0.0162 mm, and − 0.0258 ± 0.0196 mm, *P* < 0.0001, respectively; Table 2). These results indicate that the difference that occurred during 3D printing was significantly larger than the deviation that occurred after H_2_O_2_ plasma sterilisation. Therefore, the change due to H_2_O_2_ plasma sterilisation was sufficiently small to disregard.

**Table 1:** 3D comparison of 3D-CT, 3D-FDM, and 3D-GAS of the tibia models

Case	E_**FDM-CT**_ (3D-FDM vs. 3D-CT)	E_**GAS-FDM**_ (3D-GAS vs. 3D-FDM)
	RMS value (mm)	RMS value (mm)
No.1	0.1300	0.0373
No.2	0.1564	0.0568
No.3	0.1463	0.0284
No.4	0.1151	0.1123
No.5	0.1292	0.0301
No.6	0.1323	0.0320
No.7	0.0910	0.0223
No.8	0.1074	0.0258
No.9	0.1024	0.0614
No.10	0.1178	0.0402
No.11	0.1099	0.0398
No.12	0.1194	0.0311

*3D-FDM* Images obtained from reverse-scanned 3D-printed tibia models before sterilisation, *3D-CT* Original tibia images obtained from CT, *3D-GAS* Images obtained from reverse-scanned 3D-printed tibia models after sterilisation, *RMS* Root mean square

**Table 2:** Mean ± standard deviation values for comparisons of measurements obtained from 3D-CT, 3D-FDM, and 3D-GAS images of the tibial bone surfaces (*n* = 12) of six canine cadavers

Variable	E_FDM-CT_ (3D-FDM vs. 3D-CT)	E_GAS-FDM_ (3D-GAS vs. 3D-FDM)
Surface deviation		
Root mean square (mm)	0.1214 ±0.0185 ^a^	0.0431 ±0.0248
Average deviation positive (mm)	0.0973 ±0.0182 ^a^	0.0234 ±0.0162
Average deviation negative (mm)	−0.0737 ±0.0170 ^a^	− 0.0258 ±0.0196
Curvature difference of cross-sectional measurements of bone surface at:		
(a) 5% length of the tibia (mm)	0.3025 ±0.2159 ^b^	0.0491 ±0.0428
(b) 15% length of the tibia (mm)	0.0863 ±0.0227	0.0273 ±0.0180
(c) 25% length of the tibia (mm)	0.0645 ±0.0186	0.0247 ±0.0137
(d) 50% length of the tibia (mm)	0.1313 ±0.0449	0.0376 ±0.0404
(e) 75% length of the tibia (mm)	0.1307 ±0.0385	0.0263 ±0.0194
(f) 85% length of the tibia (mm)	0.1020 ±0.0353	0.0239 ±0.0160
(g) 95% length of the tibia (mm)	0.1288 ±0.0674	0.0351 ±0.0346

^a^ *P* < 0.0001.^b^ *P* < 0.0001

### Cross-sectional profile deviation of the tibia model

**Fig. 2:**
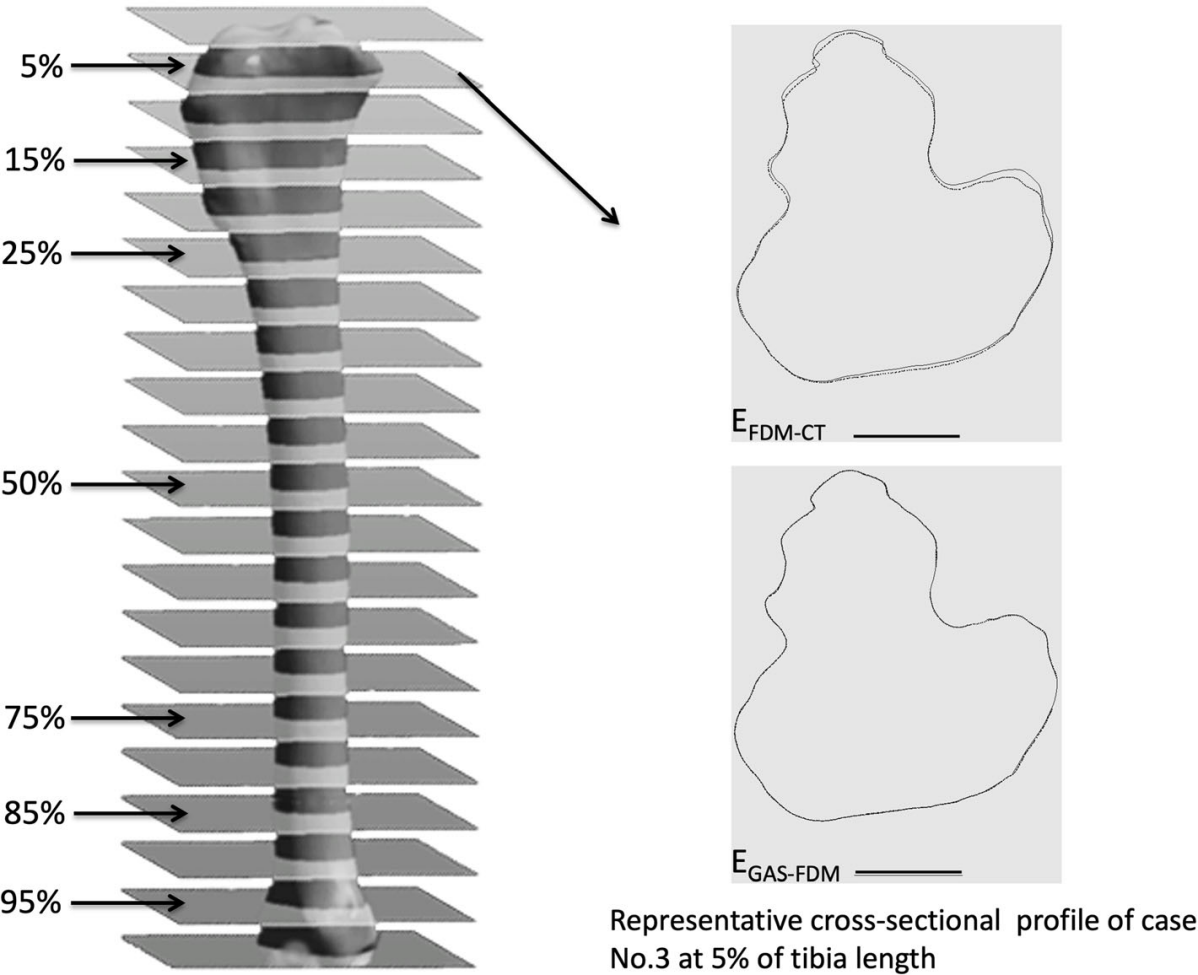
Three-dimensional tibia model and cross-sectional measurements. Total tibia length was measured between articular surfaces. The cross-sectional planes at: 5, 15, 25, 50, 75, 85, and 95% of the total tibia length were analysed (left). The map represents the difference in cross-sectional profiles of case No.3 in E_FDM-CT_ and E_GAS-FDM_ at 5% of the tibia length (right). Large deviations occurred in E_FDM-CT_ but not in E_GAS-FDM_. The dotted line denotes the test data, and the solid line denotes the reference data. Scale bar: 10 mm

**Fig. 3:**
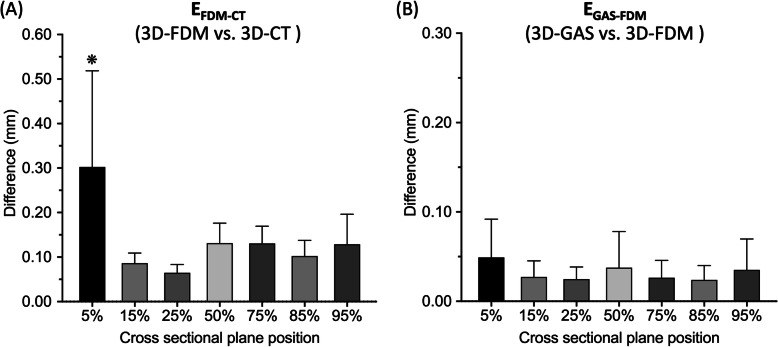
Mean RMS values at: 5, 15, 25, 50, 75, 85, and 95% of the total tibia length. **a** The mean RMS value in E_FDM-CT_ at 5% of the total tibia length was significantly higher than those of the other six cross-sections. ^❋^*P* < 0.0001. **b** The differences in mean RMS values in E_GAS-FDM_ in the seven cross-sections were not significant

Figure 2 is a graph of the E_FDM-CT_ and E_GAS-FDM_ cross-sectional profiles at 5% of the total tibia length. Additionally, the mean RMS values of E_FDM-CT_ at 5% of the total bone length (0.3025 ± 0.2159 mm) were significantly higher than those at the other six cross-sections (15%: 0.0863 ± 0.0227 mm; 25%: 0.0645 ± 0.0186 mm; 50%: 0.1313 ± 0 .0449 mm; 75%: 0.1307 ± 0.0385 mm; 85%: 0.1020 ± 0.0353 mm; and 95%: 0.1288 ± 0.0674 mm; *P* < 0.0001). The differences in mean RMS values of E_GAS-FDM_ at all seven cross-sections were not significant (Table 2 and Fig. 3). Therefore, no regional deviation was observed in the tibia model after H_2_O_2_ plasma sterilisation.

## Discussion

Some surgical studies have reported the application of 3D plastic models as surgical guides for intraoperative use [5, 6, 9, 17]. Moreover, the geometric accuracy of surgical guides is critical during surgery. Our tibia model had high bone-surface accuracy, which is beneficial for bone-plate contouring and presurgical rehearsal. Additionally, the surface curvature of the ABS 3D-printed tibia model was preserved without significant changes after gas plasma sterilisation, indicating possible clinical relevance of our model as a guide during surgery.

Our tibia model fabricated using an FDM printer and measured using a 3D point cloud had high geometric accuracy with a lower mean RMS value (E_FDM-CT_: 0.1214 mm) than those of previous studies performing linear RMS measurements on the basis of two-dimensional CT images obtained from 3D plastic long-bone models. These studies have reported RMS values of 0.40 mm [11] and < 1 mm [12]. Another study reported that a human mandible model had an absolute mean surface deviation of 0.159 mm [14], which is consistent with our observations. Given all this, it is evident that bone size can influence model accuracy. Studies have indicated dimensional errors increasing when bone model size increased to 260 mm in length [11]. They have also shown that an optimal bone model size of 120 mm in length has better linear accuracy [11, 12]. The tibial models in the present study are 150–180 mm in length and have low RMS values. Furthermore, bone surface curvature can also influence model accuracy. Two studies revealed that the largest deviation errors occurred in bone epiphyses with high levels of curvature [23, 24]. This was observed in our E_FDM-CT_ group. The curvature difference of the cross-section at the proximal tibia (5%) was significantly larger than that of the other cross-sections. Similarly, curvature variations at 15% (0.0863 mm) and 25% (0.0645 mm) of the total tibia length in E_FDM-CT_ were smaller than those in the other sections (Table 2). These variations may have occurred because tibia geometry at the proximal-medial site was relatively flat. Additionally, the small RMS error may be related to the 3D-printing build orientation with a 90° build angle and thick support structure [25, 26]. These results indicate that the data acquisition method, bone size and geometry, manufacturing process, and material could all have influenced the accuracy of the final model.

Steam heat, dry heat, H_2_O_2_ gas plasma, and ethylene oxide gas sterilisation are clinically used to sterilise plastic surgical models [1, 5]. Geometric deviation of sterilised FDM products made of ABS was mentioned in only one study [20]. Uniform rectangular samples sterilised in an autoclave that used steam heat deformed significantly (> 10 mm), and deformations could be observed by the naked eye. However, the average geometric deviation of samples after H_2_O_2_ gas plasma sterilisation was 0.036 mm [20], which is similar to our observation in E_GAS-FDM_ (0.043 mm). Our E_GAS-FDM_ results indicate acceptable mean surface deviations with no significant differences between any cross-sections, but plasma sterilisation may have a greater effect on geometric accuracy in more complex anatomical models [20].

The geometric accuracy of 3D-printed models designed for surgical use should be considered because overall errors are influenced by model geometry, printing materials, manufacturing processes, and sterilisation processes [1]. In our ABS canine tibia model, the surface deviation in E_FDM-CT_ indicated that errors occurred during the manufacturing process. These errors were significantly larger than the E_GAS-FDM_ deviations in geometric change after plasma sterilisation. These results indicate that our ABS canine tibia model was fabricated with high accuracy [14] and successfully sterilised using H_2_O_2_ plasma without significant effects on its surface geometry. However, when generating the 3D-CT model from the CT image data, some aggregate sources of errors, such as: CT scan resolution, segmentation methods, and regions of long bone may influence the accuracy of the final 3D virtual bone model. Two studies found different segmentation methods generated results with mean errors of 0.18–0.24 mm [27] and 1.07 mm [28], which are higher than the mean errors of E_FDM-CT_ (0.1214 mm) presented here. There was a variety of optimal threshold values for different regions of long bone [27]. Even using advanced methods to process and generate 3D models from CT images, the accuracy of 3D models places within the scan resolution [28]. In order to use optimal parameter settings for segmentation procedure from CT scanning to virtual model reconstruction, in the present study, we used certified commercial software^1^ that specifies accuracy is below scanning resolution and clinical relevance [29]. This means the bone models can be used for presurgical rehearsal and further clinically relevant applications in bone surgery [3, 30].

A limitation of our study was its focus on medium-sized canine tibias (length:150–180 mm) because linear RMS errors become larger with bone model size [11]. The TPLO procedure requires extensive experience and is technically challenging in small and medium-sized dogs [31]. Therefore, our tibia model may serve as a patient-specific surgical model for TPLO in medium-sized dog breeds before and during surgery. However, geometric accuracy may vary for different tibial sizes. Another limitation of the study is that we did not evaluate the consistency/reproducibility (intraobserver repeatability) of our measurement methods. It is therefore not possible to know how much of the error in our measurements results from “noise” (i.e., the error of our measurement method). If this error were to be known, it would allow for better evaluation of the true impact of H_2_O_2_ sterilization. The small RMS error in E_GAS-FDM_ suggests that our measurements were highly repeatable.

The CT-derived data were not compared with bone gross anatomy in this study. One study reported that the articular surface derived from CT images in small bones (e.g. feline radius) had dimensional errors and lacked precision when used to plan limb corrective osteotomy [32]. However, CT imaging can still be used to accurately evaluate and represent musculoskeletal anatomy [33–36] because the average deviation of CT-based human femur models from a bone-surface scan is negligible and clinically acceptable [23]. Therefore, further canine tibia cadaver reverse 3D scanning would be beneficial for ensuring geometric accuracy with respect to gross anatomy.

## Conclusion

In veterinary medicine, the increasing demand for improved visualisation and surgical outcomes have made 3D-printed anatomical models and patient-specific guides popular, and they are becoming common for implant contouring, surgery rehearsal, client communication, and education. Our study demonstrated the high geometric accuracy of an FDM-printed ABS tibia model when compared with CT data. Furthermore, surface curvature deviation of the model after H_2_O_2_ plasma sterilisation was minimal. Therefore, preoperative plate contouring and surgical planning using our tibia model may reduce surgical time and achieve adequate bone reproduction. Additionally, our model may be used during operations because it has nearly the same surface curvature as that of real bone. Additional studies evaluating the geometric accuracy of patient-specific 3D-printed cutting guides for intraoperative use would be useful as they will help improve assessment of models used in a clinical setting.

## Methods

### Sample collection

All experimental protocols complied with the guidelines for the care and use of animals and were approved by the Institutional Animal Care and Use Committee of National Taiwan University (NTU108–EL-00015). Six mixed-breed skeletally mature canine cadavers (Mean body weight: 12 kg (10–15); 3 female and 3 male) were used and informed consent obtained from the animal shelter. The dogs’ deaths were unrelated to this study. The dogs had no known histories of orthopaedic surgery. Coxofemoral amputation was performed on all 6 cadavers, and the 12 pelvic limbs were covered with saline-rinsed gauze and stored at − 20 °C. All experiments were performed 24 h after the pelvic limbs were thawed at room temperature.

### 3D-printed tibia model

To obtain a 3D reconstruction, all the limbs underwent CT [1] imaging on a 128-slice CT scanner (Ingenuity Core 128, Philips Medical Systems, Cleveland, OH, USA). All limbs were placed in a caudal extension position with tibial diaphysis being perpendicular to the plane of imaging. Scanning was conducted at 120 kV and 105–115 mA [10]. The scanning procedure was performed using 0.5 mm CT slices (resolution 512 × 512 pixels, pixels spacing = 0.43 mm/pixels) around the whole limb. Image preprocessing was performed following the procedure given by the commercial software^1^: First, we used a threshold value at 220–3000 HU to separate bone and soft tissue. Segmentation was performed to extract the tibia. The 3D tibial model was reconstructed using a high-frequency algorithm (Marching Cubes), and then smoothing performed. Finally, the 3D tibial model was exported as standard tessellation language (STL)-format files.

Both tibias of each dog were printed to be the same size as the cadavers’ tibias. The tibia models were printed on a 3D printer. The printing material was ABS with a layer height of 0.2 mm. Additive manufacturing error relates to layer height at 0.2 mm (Up Box+, Milpitas, CA, USA). All tibia models were printed with the long-bone axis parallel to the printer surface and the proximal-medial side facing up with a substantial ABS-printed support structure underneath. After printing, the ABS base structure was gently removed manually from the tibia bone models. There was no further postprocessing performed on the tibial bone models.

### Tibia morphological analysis before and after sterilisation

After careful harvesting of the 12 tibia models, reverse-engineering technology was used to analyse their errors [37]. The tibias were first scanned using an optical scanner (Breuckmann smartSCAN 3D, AICON 3D Systems, Braunschweig, Germany) with an accuracy of 0.02 mm, and 3D virtual (point cloud) models (3D-FDM) were obtained from the images of the scanned 3D-printed tibia models. The scanned images were stacked and decomposed into multiple points to form the test model. The virtual model (3D-CT) was obtained from the original tibia CT images and decomposed into multiple points to form the reference model. Subsequently, the differences between the 3D-FDM and 3D-CT models were compared on a point-by-point basis by superimposing the two virtual models with software^2^, and calculations were performed using the closest projection point algorithm and Newton’s method [38, 39]. A 2-phase superimposing process was performed: an initial manual labelling of bony structures on tibial bone models was followed by a second automated registration. Additionally, root mean square (RMS) errors were calculated. RMS errors are generally used to assess the mean value of errors and are typically used as a criterion for assessing the similarity of two N-dimensional vector sets after optimal superimposition [40]. A higher RMS value indicates a larger error. The superimposition results were also illustrated by a colour difference map (Fig. 1a). The maximum and minimum values of the colour difference map were + 0.5000 and − 0.5000 mm, respectively.

A low-temperature H_2_O_2_ STERRAD NX steriliser (50 min/cycle, temperature < 55 °C, Johnson & Johnson Company) was used to sterilise the printed tibia models. The sterilised tibias were scanned a second time, and 3D virtual models (3D-GAS) were reconstructed. The superimposition results were also illustrated by a colour difference map (Fig. 1b). The maximum and minimum values of the colour difference map were + 0.2000 and − 0.2000 mm_,_ respectively. The difference between the 3D-GAS and 3D-FDM was also evaluated using the closest projection point algorithm and Newton’s method [38, 39]. Both the manufacturing process and sterilization can influence geometric accuracy in printed tibial models. We wanted to identify which of them impacted tibial models the most. Consequently, two comparisons were made: E_FDM-CT_ (3D-FDM vs. 3D-CT) and E_GAS-FDM_ (3D-GAS vs. 3D-FDM). Reverse-engineering software^2^ was employed for error analysis among all models.

### Morphological analysis at the tibia cross-sectional profile

Following the previous mentioned superimposition of the two virtual models (Fig. 1), tibia length was defined as the longest distance from the proximal to distal end in the craniocaudal view of the 3D virtual model. The cross-sectional plane was simulated on the 3D virtual model at: 5, 15, 25, 50, 75, 85, and 95% of the previously recorded tibia length, and the contour points where the tangential plane intersected the model were extracted as point cloud data. A total of seven cross-sectional datasets were obtained for each tibia model and the RMS value was calculated through comparison of E_FDM-CT_ and E_GAS-FDM_ (Fig. 3, Table 2).

### Statistical analysis

A Mann-Whitney U test was conducted to evaluate the RMS mean differences between E_FDM-CT_ and E_GAS-FDM_. A one-way analysis of variance was used to test the RMS mean differences among the seven cross-sectional datasets in each group. All statistical analyses were performed using GraphPad Prism 8.0 (GraphPad Software, Inc., San Diego, CA, USA). The significance level was set at *P* < 0.05.

## Data Availability

All data generated or analyzed during this study are included in this published article and available from the corresponding author on reasonable request.

## Abbreviations

3D: Three-dimensional
ABS: Acrylonitrile butadiene styrene
FDM: Fused deposition modelling
H_2_O_2_: Hydrogen peroxide
RMS: Root mean square
TPLO: Tibial plateau levelling osteotomy
CT: Computed tomography
STL: Standard tessellation language

## Footnotes

1 Mimics 22.0, Materialise NV, Belgium.

2 RevCAD, PouYuen Tech Corp., Taoyuan, Taiwan.
